# Perinatal Maternal Administration of *Lactobacillus paracasei* NCC 2461 Prevents Allergic Inflammation in a Mouse Model of Birch Pollen Allergy

**DOI:** 10.1371/journal.pone.0040271

**Published:** 2012-07-06

**Authors:** Irma Schabussova, Karin Hufnagl, Mimi L. K. Tang, Elisabeth Hoflehner, Angelika Wagner, Gerhard Loupal, Sophie Nutten, Adrian Zuercher, Annick Mercenier, Ursula Wiedermann

**Affiliations:** 1 Institute of Specific Prophylaxis and Tropical Medicine, Center for Pathophysiology, Infectiology and Immunology, Medical University of Vienna, Vienna, Austria; 2 Department of Allergy and Immunology, Royal Children’s Hospital, The University of Melbourne, Melbourne, Australia; 3 Department of Allergy and Immune Disorders, Murdoch Children’s Research Institute, Melbourne, Australia; 4 Department of Paediatrics, Royal Children’s Hospital, The University of Melbourne, Melbourne, Australia; 5 Department of Pathobiology, Institute of Pathology and Forensic Veterinary Medicine, The University of Veterinary Medicine Vienna, Vienna, Austria; 6 Nutrition and Health Department, Nestlé Research Center, Lausanne, Switzerland; 7 CSL Behring AG, Bern, Switzerland; Centre de Recherche Public de la Santé (CRP-Santé), Luxembourg

## Abstract

**Background:**

The hygiene hypothesis implies that microbial agents including probiotic bacteria may modulate foetal/neonatal immune programming and hence offer effective strategies for primary allergy prevention; however their mechanisms of action are poorly understood. We investigated whether oral administration of *Lactobacillus paracasei* NCC 2461 to mothers during gestation/lactation can protect against airway inflammation in offspring in a mouse model of birch pollen allergy, and examined the immune mechanisms involved.

**Methods:**

BALB/c mice were treated daily with *L. paracasei* in drinking water or drinking water alone in the last week of gestation and during lactation. Their offspring were sensitized with recombinant Bet v 1, followed by aerosol challenge with birch pollen extract.

**Results:**

Maternal exposure to *L. paracasei* prevented the development of airway inflammation in offspring, as demonstrated by attenuation of eosinophil influx in the lungs; reduction of IL-5 levels in bronchoalveolar lavage, and in lung and mediastinal lymph node cell cultures; and reduced peribronchial inflammatory infiltrate and mucus hypersecretion. While allergen-specific IgE and IgG antibody levels remained unchanged by the treatment, IL-4 and IL-5 production in spleen cell cultures were significantly reduced upon allergen stimulation in offspring of *L. paracasei* treated mice. Offspring of *L. paracasei* supplemented mothers had significantly reduced Bet v 1-specific as well as Concanavalin A-induced responses in spleen and mesenteric lymph node cell cultures, suggesting the modulation of both antigen-specific and mitogen-induced immune responses in offspring. These effects were associated with increased Foxp3 mRNA expression in the lungs and increased TGF-beta in serum.

**Conclusion:**

Our data show that in a mouse model of birch pollen allergy, perinatal administration of *L. paracasei* NCC 2461 to pregnant/lactating mothers protects against the development of airway inflammation in offspring by activating regulatory pathways, likely through TLR2/4 signalling.

## Introduction

The prevalence of allergic diseases in industrialized countries has increased rapidly over the past decades. The reasons for the steady increase are not yet fully understood. While genetic factors contribute to increased disease risk [1], [2], genetic predisposition and heritable factors cannot solely explain the rise in allergies. Rather, environmental factors such as reduced microbial stimulation of the immune system in infancy as a consequence of improved hygiene seem to play important roles. However, not only has the extrinsic microbial environment changed, but so has the composition of intestinal microbiota possibly due to differences in diet or over-use of antibiotics [3], [4]. Several studies have shown differences in the composition of gut microbiota between allergic and non-allergic children [5], [6], [7], [8] and reduced diversity of the infant’s intestinal flora was associated with increased risk of allergic sensitization [9]. In a prospective study, children who later developed allergic disease were less often colonized with bifidobacteria during the first year of life [10]. Moreover, atopic sensitization was correlated with a reduced ratio of faecal bifidobacteria to clostridia in the early perinatal period [6]. These findings provide a rationale for use of probiotic bacteria to prevent allergic disease.

There is evidence that events occurring in the first year of life and even before delivery have the potential to program persisting immunological phenotypes that determine the subsequent risk of allergic disease [11], [12]. Thus, pre-, peri-, and/or postnatal interventions offer a promising approach to modulate immune responses and promote a non-allergic status. Moreover, interventions during pregnancy/lactation might have considerable advantages in terms of convenience and compliance compared to child-directed interventions. Indeed, preclinical and clinical studies have shown that perinatal interventions with specific probiotic bacteria can mediate protection against infant allergic diseases [13].

In animal studies, we have shown previously that probiotic bacteria might regulate the allergic phenotype through a number of different pathways including: (i) induction of Th1-type immunity [14]; (ii) generation of regulatory responses [15], [16]; (iii) and production of IgA [14]. Using a mouse model of allergic poly-sensitization, we recently demonstrated that *Lactobacillus paracasei* NCC 2461, a strain with the capacity to induce Th1 and regulatory responses *in vitro* [17], applied at the time of sensitization and challenge significantly suppressed airway inflammation and down-regulated allergen-specific immune responses [15]. In the present study we show that maternal supplementation with *L. paracasei* NCC 2461 protects against the development of allergic airway inflammation in offspring, likely mediated by induction of regulatory responses.

## Materials and Methods

### Animals

Pregnant BALB/c mice (day 14 of pregnancy) were purchased from Charles River (Sulzfeld, Germany) and maintained under conventional housing conditions. Female TLR2- and TLR4-deficient and wild type mice with C57BL/6 genetic background were obtained from M. Müller (Vienna, Austria).

### Ethics Statement

All experiments were approved by the Animal Experimentation Committee of the Medical University of Vienna and by the Federal Ministry of Science and Research (BMWF-66.009/0175-C/GT/2007).

### Probiotic Bacteria

Probiotic strain *L. paracasei* NCC 2461 (CNCM I-2116, hereafter *L. paracasei*) was provided by Nestlé Research Center (Lausanne, Switzerland). For perinatal oral administration, spray-dried bacterial preparation was diluted in drinking water at 5×10^8^ CFU/ml. For *in vitro* experiments, *L. paracasei* preparation was inactivated with 1% formaldehyde-PBS for 3 h at room temperature, washed twice with sterile PBS, and stored at –20°C.

### Perinatal *L. paracasei* Exposure

**Figure 1:**
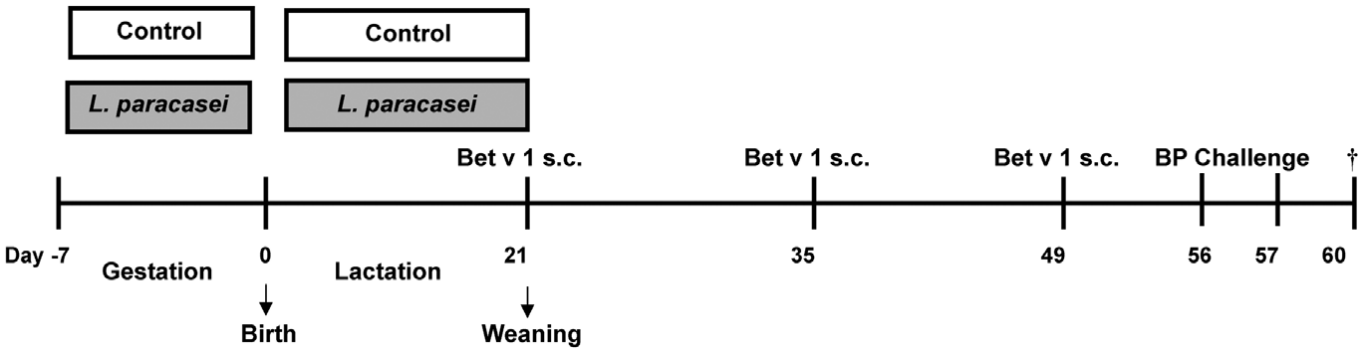
Experimental design. Pregnant mice were fed daily with *L. paracasei* in drinking water during the last week of gestation and lactation. Control mothers received drinking water without *L. paracasei*. Offspring mice were immunized subcutaneously (s.c.) with Bet v 1 on days 21, 35, and 49, then aerosol challenged with birch pollen (BP) on two consecutive days 1 week after the last s.c. sensitization (days 56, 57), and sacrificed on day 60.

Based on preliminary studies of dosage and timing of *L. paracasei* application (data not shown), pregnant BALB/c mice (mothers; n = 5/group; two independent experiments were performed) were treated with an average daily dose of 2×10^9^ CFU *L. paracasei/*mouse, starting from the third trimester of gestation (day -7) and continuing throughout the lactation period until the day of the weaning (day 21). Age-matched control animals received water only (Experimental design; Fig 1).

### Mouse Model of Birch Pollen Induced Allergic Airway Inflammation

A validated model of birch pollen induced allergic airway inflammation [18] was applied in offspring from *L. paracasei*-exposed or control-treated mother mice. Briefly, offspring (n ≥5/group) was sensitized with recombinant Bet v 1 (Biomay, Vienna, Austria) emulsified in aluminium hydroxide (alum, Serva, Heidelberg, Germany) subcutaneously (s.c.) (1 µg in 150 µl on days 21, 35, 49); and then challenged with aerosolized 1% birch pollen extract (BP; Allergon, Välinge, Sweden) 1 week after the last sensitization on days 56–57 (Fig 1). Mice were terminally anesthetized 72 h after final airway challenge (day 60) [18].

### Bronchoalveolar Lavage and Differential Cell Counts

Offspring from *L. paracasei*- or control-treated mother mice were terminally anesthetized and bronchoalveolar lavage (BAL) was performed two times using 2 ml PBS containing protease inhibitor cocktail (Roche, Mannheim, Germany). Total leukocytes were counted and cytospins (Shadon Cytospin®, Shadon Southern Instruments, USA) were stained with hematoxylin and eosin (H&E; Hemacolor®, Merck, Darmstadt, Germany) for differential cell counts (200 cells were counted per cytospin). Cell-free supernatants were stored at –20°C for further analysis.

### Lung Histology

Whole lungs were fixed with 10% formaldehyde-PBS and paraffin-embedded. Tissue sections were stained with H&E. Airway mucus occlusion was analyzed on periodic acid-Schiff-stained (PAS, Sigma-Aldrich) sections.

### IgG1, IgE and IgA Antibody Levels in BAL, Serum and Gut Samples

Blood samples were taken by tail bleeding on the day of sacrifice and sera were stored at –20°C. Small intestine was excised and faeces removed by flushing the lumen with 2 ml PBS. The intestine was cut open lengthwise and frozen in 1 ml of protease inhibitor cocktail. After thawing, samples were incubated in 20% saponine solution (Sigma-Aldrich) overnight to permeabilize cell membranes. Supernatants were collected after centrifugation (2000 g; 10 min) and stored at –20°C. Levels of serum anti-Bet v 1 IgG1 and IgE and total levels of gut IgA were measured by ELISA. For the detection of antigen-specific antibodies, microtitre plates (Nunc, Wiesbaden, Germany) were coated with Bet v 1 (2 µg/ml). Serum samples were diluted 1/1000 for IgG1 and 1/10 for IgE. For the detection of total IgA, plates were coated with anti-IgA (2 µg/ml; Pharmingen, San Diego, CA). BAL fluid was applied neat and gut lavages were diluted 1/2500. Rat anti-mouse IgG1, IgE and IgA antibodies (1/500; Pharmingen, San Diego, CA) were applied and peroxidase-conjugated mouse anti-rat IgG antibodies (1/1000; Jackson, Immuno Labs., West Grove) were used for the detection. Data represent mean values ± SEM of optical density (OD) values from duplicate wells.

### *Ex vivo* Stimulation of Cell Cultures

Spleens, lungs, mediastinal lymph nodes, and mesenteric lymph nodes (MLN) were collected on sacrifice and single-cell suspensions were prepared from each organ. Where indicated, cells (2.5×10^6^/ml) were stimulated with Bet v 1 (10 µg/ml), Concanavalin A (ConA) (1 µg/ml) or media alone in 96-well plates at 37°C for 72 h in culture medium (RPMI 1640 supplemented with 10% heat-inactivated FCS, 2 mM L-glutamine, 100 U/ml penicillin, 100 µg/ml streptomycin). Proliferative responses of spleen and MLN cell cultures were determined by scintillation counting after addition of ^3^[H]-thymidine (0.5 µCi/well; Amersham, Buchter, Braunschweig, Germany) for the last 18 h. In addition, spleen cells from offspring of sham-treated mothers were incubated with *L. paracasei* (10^7^ CFU/ml) admixed to Bet v 1 (10 µg/ml) for 48 h.

### Cytokine Measurements

Levels of IL-4, IL-5, IL-10, and TGF-β were measured by ELISA (Endogen, Cambridge, MA) according to the manufacturer’s instructions. Levels of IFN-γ were measured as previously described [19].

### Quantification of Foxp3 mRNA Expression in Offspring Lung by Real-time RT-PCR

Total RNA was extracted from lung tissue of offspring from *L. paracasei*- or control-treated mothers as described previously [18]. The housekeeping gene *5-aminolevulinic acid synthase 1* (ALAS1) (Universal ProbeLibrary probe #64; Roche) was used to standardize the amount of sample cDNA. Data are presented as the relative ratio of the target gene to ALAS1.

### Stimulation of TLR Knockout Spleen Cells

Splenocytes from C57BL/6 wild type, TLR2- and TLR4-knockout mice (2.5×10^6^/ml) were stimulated with *L. paracasei* (10^7^ CFU/ml), LPS (*E. coli* 0111:B4; 1 µg/ml; InvivoGen; TLR4 ligand), Pam (Pam_3_CSK4, TLR2 ligand; 1 µg/ml; InvivoGen), or media alone for 48 h.

### Generation and Stimulation of Bone Marrow Derived Dendritic Cells

Bone marrow derived dendritic cells (BM-DC) were generated as previously described [20]. Briefly, the bone marrow precursors were isolated from femurs and tibias of BALB/c mice. Cells were cultured at 2×10^5^/ml in bacteriological Petri dishes in 10 ml culture medium with GM-CSF (20 ng/ml; Sigma-Aldrich). Fresh medium was added at days 3 and 6 and BM-DC were used on day 10 of culture. BM-DC were stimulated with *L. paracasei* at a final concentration of 10^6^ or 10^7^ CFU/ml for 24 h, after which supernatants were tested by ELISA. LPS (1 µg/ml) was used as a positive control.

### Statistical Analysis

All data are shown as mean ± SEM. Significance was analyzed using the non-parametric Mann-Whitney U-test (Graph Prism; Graph Pad Software, Inc, San Diego, CA). Significant differences were considered at p<0.05.

## Results

### Maternal Perinatal *L. paracasei* Application does not Influence Sensitization to Bet v 1 in Offspring

In a mouse model of birch pollen allergy (Fig 1), sensitization and challenge of offspring induced allergen-specific antibody production. Maternal application of *L. paracasei* did not hinder this process, and anti-Bet v 1 IgG1 and IgE antibody titres in serum did not differ between offspring of *L. paracasei*-treated and control mice (IgG1: 1.12±0.16 vs. 1.65±0.17, p = 0.76; IgE: 0.58±0.12 vs. 0.61±0.14, p = 0.89; n ≥8 per group; data from 3 separate experiments which each yielded similar results).

### Maternal Perinatal *L. paracasei* Supplementation Protects Offspring from Development of Allergic Airway Inflammation

*L. paracasei* was detected by PCR analysis using strain specific primers exclusively in faecal samples from *L. paracasei*-treated, and not from control mothers (data not shown). Importantly, *L. paracasei* was not detected in offspring of either *L. paracasei*-treated or control mothers (data not shown).

**Figure 2:**
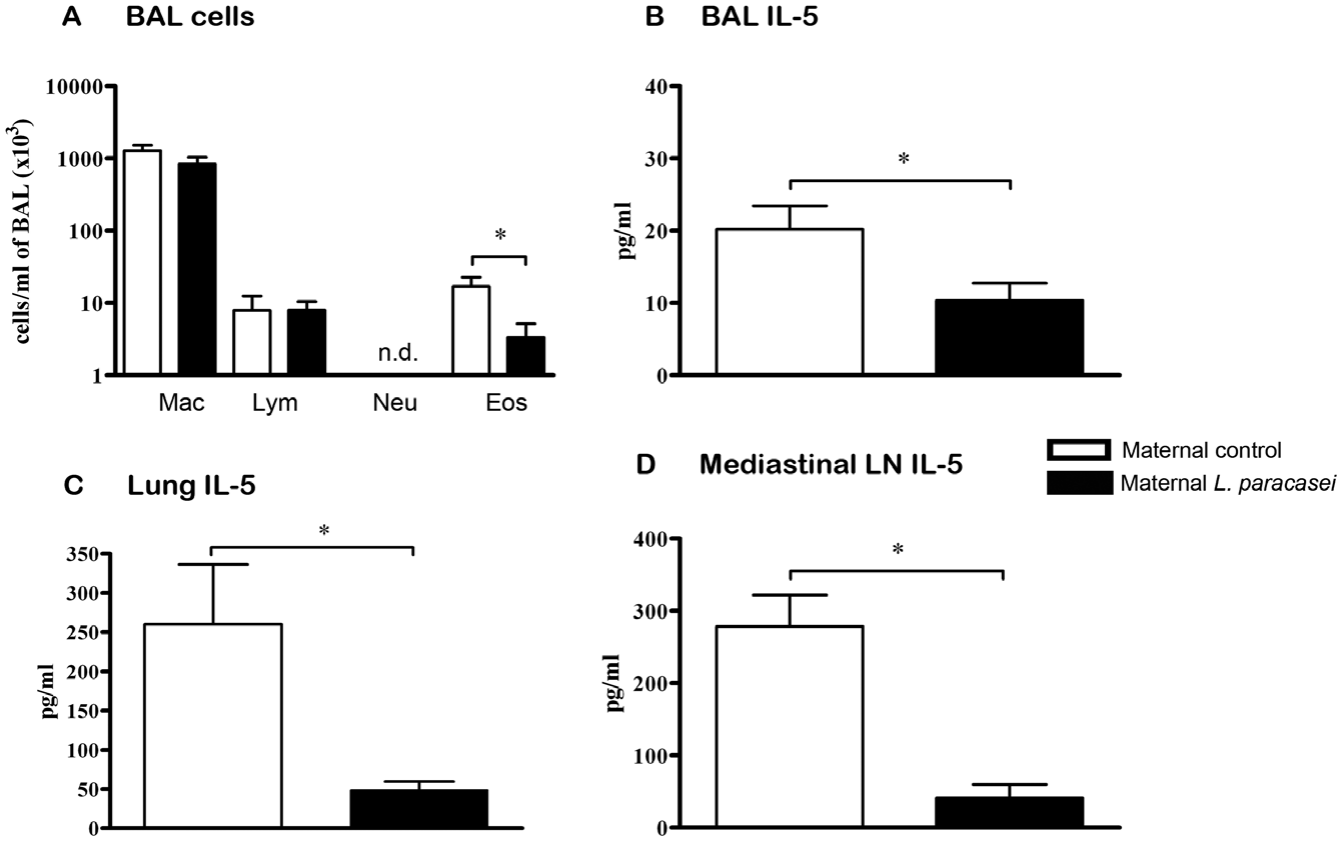
Maternal L. paracasei supplementation protects offspring from development of allergic responses. (A) Differential leukocyte numbers in BAL fluid from offspring of *L. paracasei*-treated mothers or controls. Levels of IL-5 in BAL fluid (B) and production of IL-5 in Bet v 1-re-stimulated lung (C) and mediastinal lymph node (D) cell cultures were assessed by ELISA. Data is represented as mean ± SEM (n ≥8; results represent pooled data from two independently performed experiments). *p<0.05; n.d. not detected. Mac = macrophages, Lym = lymphocytes, Neu = neutrophils, Eos = eosinophils.

**Figure 3:**
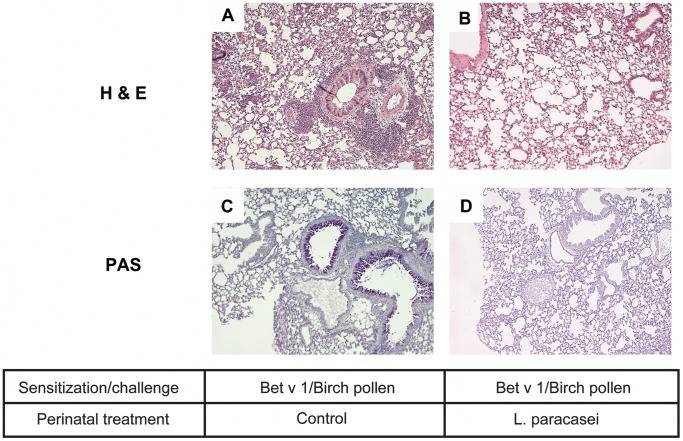
Maternal L. paracasei supplementation reduces airway inflammation in offspring. Representative lung tissue sections from sensitized and challenged offspring of *L. paracasei*-treated (B, D) or sham-treated (A, C) mothers. Samples were stained with hematoxylin and eosin (A, B) to assess inflammation/cellular infiltration or with periodic acid-Schiff stain (PAS) (C, D) to enumerate mucus-producing goblet cells. Magnification 100 x.

Perinatal administration of *L. paracasei* to mothers significantly inhibited the development of airway inflammation in their progeny. Numbers of BAL eosinophils were significantly reduced in sensitized/challenged offspring from *L paracasei*-treated mothers as compared to sensitized/challenged offspring from sham-treated control mothers (Fig 2*A*). Offspring of *L. paracasei*-exposed mothers showed considerably reduced peribronchial inflammation with only rare mononuclear cell infiltrates (Fig 3*B*) as compared to offspring of control mice that showed massive cellular infiltration around bronchi and blood vessels (Fig 3*A*). In addition, goblet cell hyperplasia and mucus hypersecretion were strongly reduced in offspring of *L. paracasei*-treated mothers as compared to the control group (Fig 3*C-D*). Furthermore, levels of the IL-5 in BAL fluid (Fig 2*B*), as well as lung tissue (Fig 2*C*) and mediastinal lymph node (Fig 2*D*) cell culture supernatants from offspring of *L. paracasei*-exposed mothers were significantly reduced compared with that from offspring of sham-treated controls.

### Maternal *L. paracasei* Supplementation Results in Up-regulation of Foxp3 in Offspring Lung Tissues

**Figure 4:**
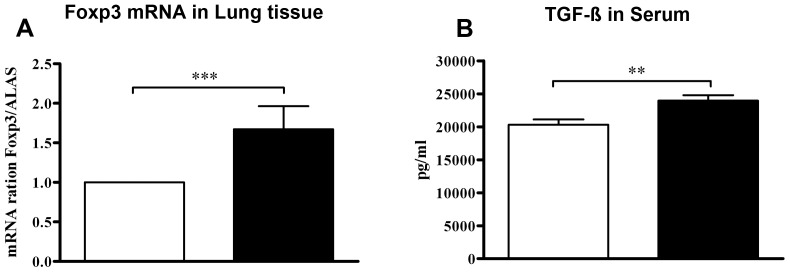
Maternal L. paracasei supplementation results in up-regulation of regulatory markers. (A) Expression of Foxp3 mRNA in lung tissue from offspring of *L. paracasei*-treated or sham-treated mothers. Foxp3 mRNA data is expressed relative to the housekeeping gene ALAS1. (B) Serum levels of TGF-β were determined by ELISA. Data is expressed as mean ± SEM (n ≥8; results represent pooled data from two independently performed experiments). **p<0.001; ***p<0.0001.

Since we observed marked suppression of airway inflammation and IL-5 responses in the lungs of offspring from *L. paracasei-*treated mothers, we examined levels of Foxp3 mRNA expression in this organ. Offspring of *L. paracasei-*treated mothers exhibited increased expression of Foxp3 mRNA in comparison to controls (Fig 4*A*). Additionally, offspring of mothers exposed to *L. paracasei* had markedly increased serum levels of the regulatory cytokine TGF-β as compared to offspring from control mothers (Fig 4*B*). As TGF-β is an important switch factor for B cells to produce IgA antibodies [21] and a potential regulatory role of IgA against allergic disease has been suggested [22] we examined IgA production in offspring. We found that maternal *L. paracasei* treatment had no effect on total IgA levels in BAL from offspring (0.9±0.2 OD vs. 1.0±0.2 OD, p = 0.678; offspring from perinatal *L. paracasei* vs. offspring from perinatal sham, respectively; n≥5 per group; data from 2 separate experiments which each yielded similar results). However, offspring from *L. paracasei* treated mothers had increased levels of total gut IgA when compared to control mice (2.1±0.0 OD vs. 1.9±0.0 OD, p = 0.003; offspring from perinatal *L. paracasei* vs. offspring from perinatal sham, respectively; n≥5 per group; data from 2 separate experiments which each yielded similar results).

### Maternal *L. paracasei* Supplementation Inhibits Systemic Allergen-specific Recall Responses in Offspring

**Figure 5:**
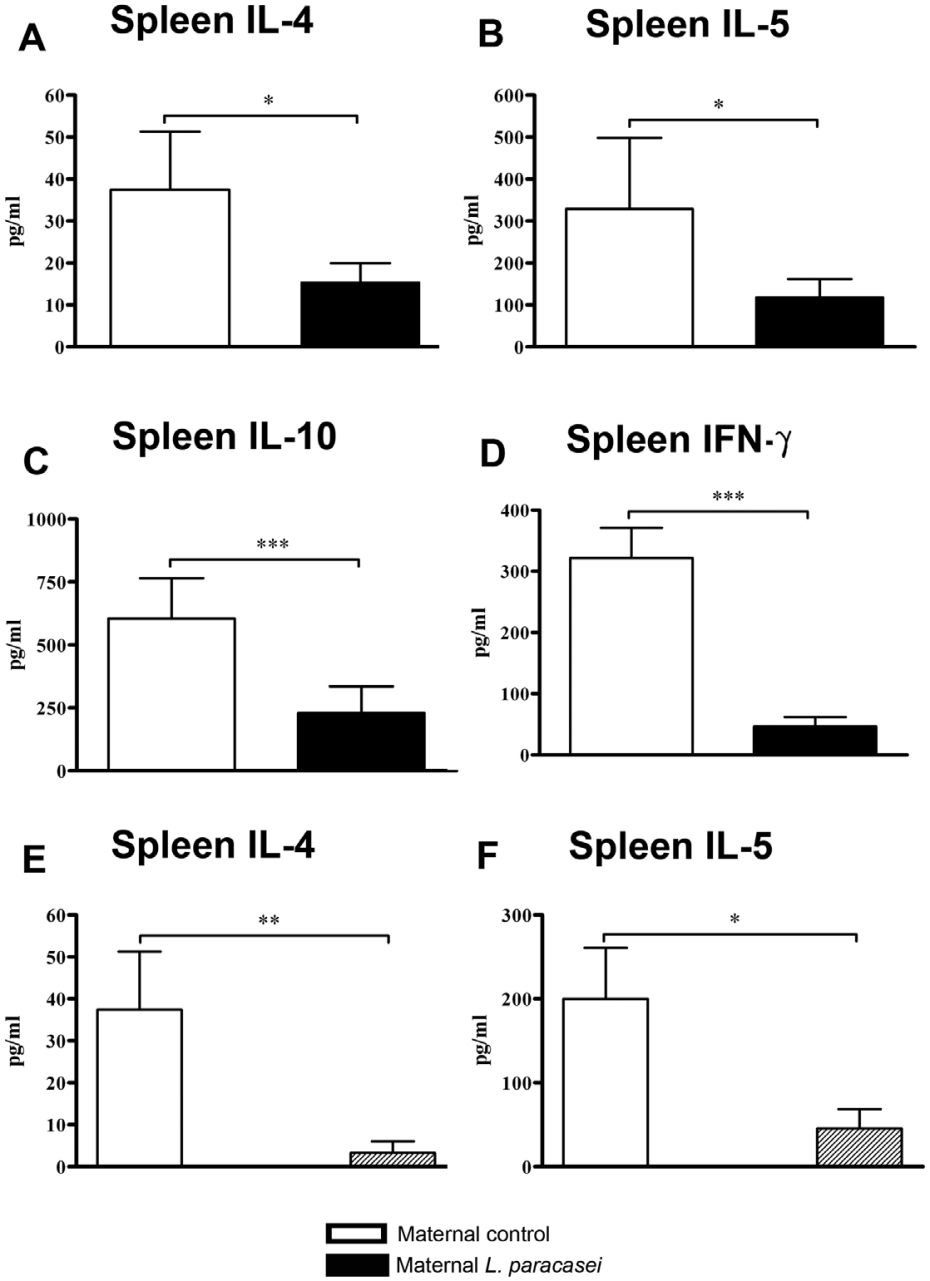
L. paracasei inhibits allergen-specific recall responses in offspring both in vivo and in vitro. (A-D) Splenocytes from Bet v 1 sensitized/challenged offspring of *L. paracasei*-treated or sham-treated mothers were stimulated with Bet v 1 (A-D). Splenocytes from sensitized/challenged offspring of sham-treated mothers were stimulated with Bet v 1 (Bet), *L. paracasei* (*L.para*) or Bet v 1/*L. paracasei* (Bet/*L.para*) (E, F). Cytokine production was determined by ELISA. Data is expressed as mean ± SEM (n ≥8; results represent pooled data from two independently performed experiments). *p<0.05; **p<0.01; ***p<0.001; n.d. not detected. For *in vitro* stimulation, *L. paracasei* NCC 2461 was treated with formaldehyde.

To test whether the effects on airway inflammation in offspring were associated with changes in distal immune responses, allergen-specific responses were examined in offspring splenocyte and MLN cultures. Production of IL-4 and IL-5 in Bet v 1-stimulated splenocyte cultures was significantly lower in the offspring of *L. paracasei*-supplemented mothers compared with offspring of control mothers (Fig 5*A-B*). Interestingly, production of the regulatory cytokine IL-10 (Fig 5*C*) and the Th1 cytokine IFN-γ (Fig 5*D*) was also markedly lower in offspring of *L. paracasei*-treated mothers as compared to offspring of controls. Furthermore, IL-4 and IFN-γ secretion by Bet v 1-stimulated MLN cells from offspring of *L. paracasei* treated mothers were significantly lower than for offspring of control mothers (IL-4: 1.8±0.5 pg/ml vs. 6.0±2.0 pg/ml, p = 0.02; and IFN-γ: 73.5±19.0 pg/ml vs. 220.7±64.8 pg/ml, p = 0.03; perinatal *L. paracasei* vs. perinatal sham, respectively; n ≥4 per group; data from 2 separate experiments which each yielded similar results). We further assessed whether *L. paracasei* might have similar effects on allergen-specific cytokine production *in vitro.* Splenocytes from offspring of sham-treated mothers were cultured with Bet v 1 in the presence or absence of *L. paracasei.* Bet v 1-induced IL-4 and IL-5 production was significantly reduced in the presence of *L. paracasei* (Fig 5*E-F*).

### Maternal *L. paracasei* Supplementation Inhibits Mitogen-induced Cytokine Responses in Offspring

**Figure 6:**
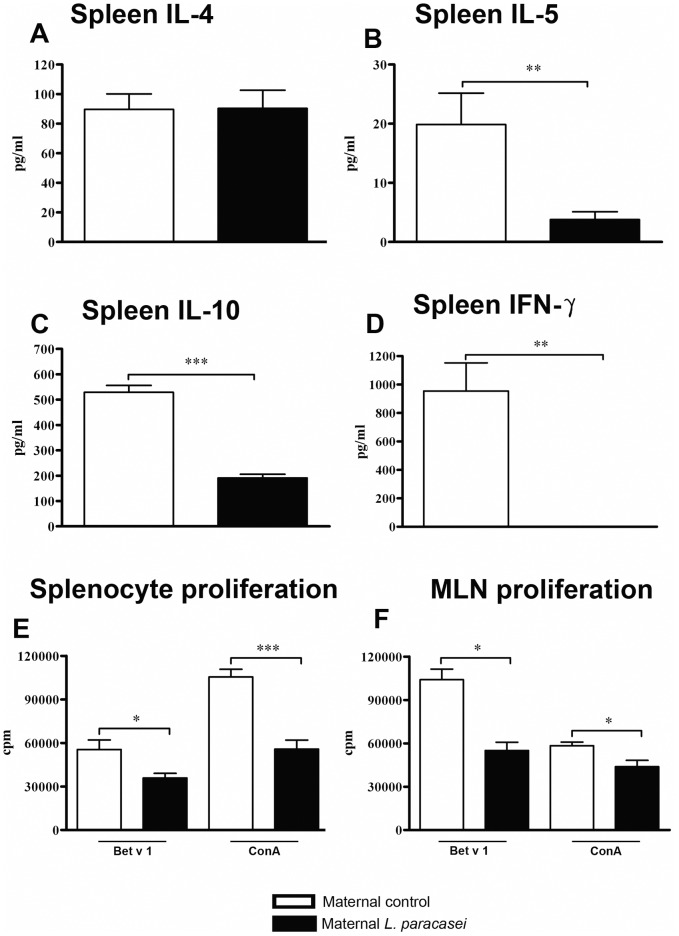
Maternal L. paracasei supplementation reduces mitogen-induced immune responses. (A-D) Splenocytes from offspring of *L. paracasei*-treated or sham-treated mothers were stimulated with ConA and cytokine production assessed by ELISA. Proliferation of splenocytes (E) and MLN cells (F) following stimulation with Bet v 1 or ConA was measured by ^3^[H]-thymidine incorporation. Data is expressed as mean ± SEM. Spleen: n≥8 per group; MLN: n ≥3 per group; (results represent pooled data from two independently performed experiments). *p<0.05; **p<0.001; ***p<0.0001.

Having determined that perinatal *L. paracasei* supplementation reduced allergen-specific immune responses in offspring, we aimed to assess whether mitogen-induced immune responses were influenced as well. Indeed, ConA-stimulated splenocytes from offspring of *L. paracasei*–supplemented mothers had significantly reduced secretion of IL-5, IL-10, and IFN-γ in comparison to splenocytes from offspring of control mice (Fig 6*B-D*). Similarly, we observed a trend towards reduced secretion of Th1- and Th2-related cytokines by ConA-stimulated MLN cells in offspring of *L. paracasei*-treated mothers in comparison to offspring of sham-treated controls (IL-4: 6.3±1.1 pg/ml vs. 10.2±2.0 pg/ml, p = 0.23; and IFN-γ: 281.6±93.5 pg/ml vs. 451.8±133.5 pg/ml, p = 0.35; perinatal *L. paracasei* vs. perinatal sham, respectively; n ≥3 per group; data from 2 separate experiments which each yielded similar results).

### Maternal *L. paracasei* Supplementation Inhibits Allergen- and Mitogen-induced Proliferative Responses of Spleen and MLN Cells in Offspring

Remarkably, maternal *L. paracasei*-supplementation led to reduced allergen-induced and mitogen-induced proliferative responses of spleen and MLN cells from offspring *ex vivo* (Fig 6*E-F*).

### *L. paracasei* Acts through TLR2 and TLR4 to Induce Production of Regulatory Cytokines by Splenocytes

**Figure 7:**
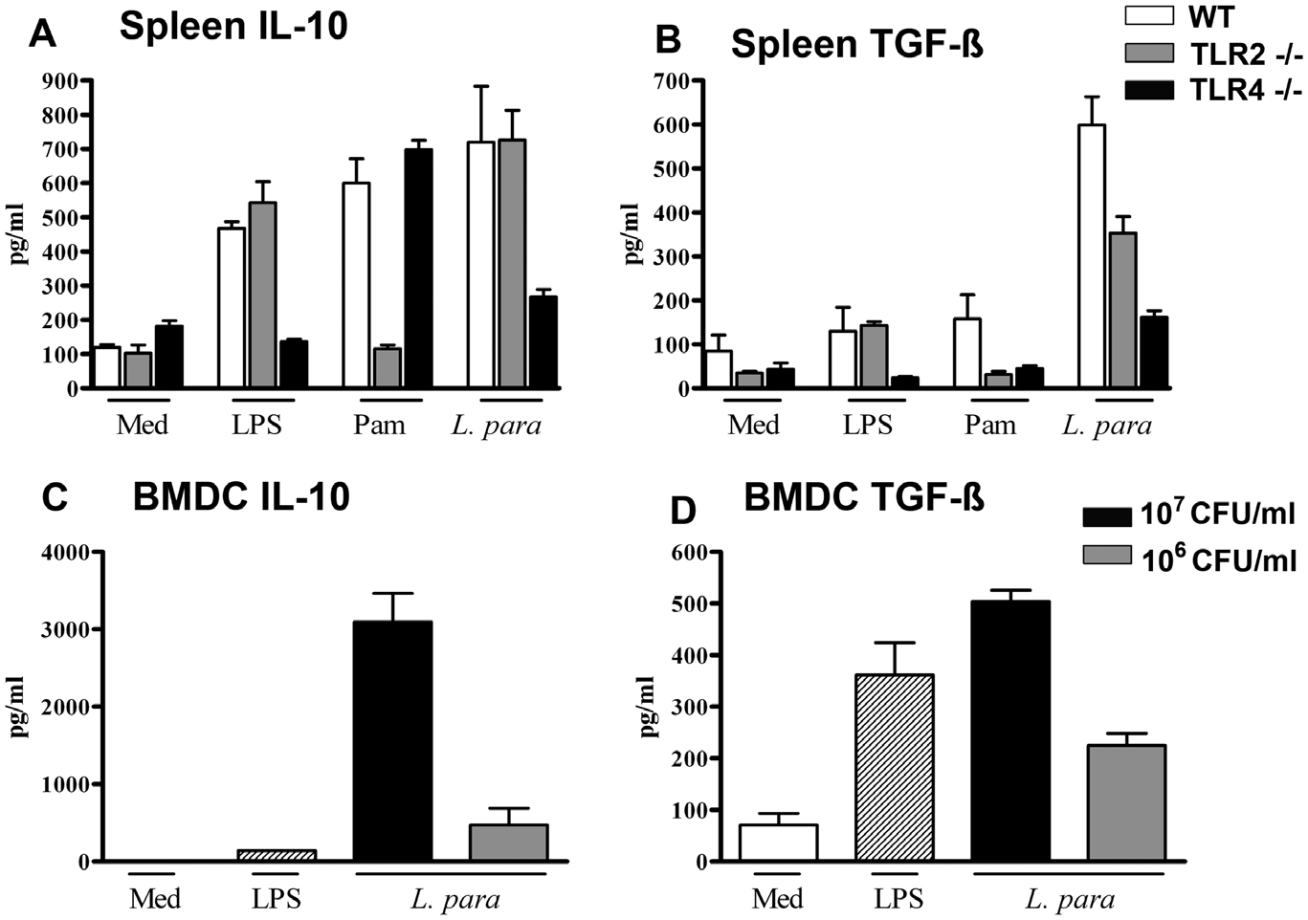
L. paracasei mediates the production of regulatory cytokines through TLRs pathways. (A, B) Splenocytes from wild-type (WT), TLR2^−/−^, or TLR4^−/−^ mice were stimulated with *L. paracasei*, LPS or Pam3CSK4 (Pam). Untreated cells (Med) served as a negative control. (C, D) BM-DC were stimulated with *L. paracasei* or LPS. Cytokine production was assessed by ELISA. Data is expressed as mean ± SEM; (results represent pooled data from two independently performed experiments). For *in vitro* stimulation, *L. paracasei* was treated with formaldehyde.

We have shown previously that intranasal application of *L. paracasei* led to increased mRNA expression of TLR2 and TLR4 receptors in draining lymph nodes [15]. We therefore investigated whether TLR2 or TLR4 are functionally involved in mediating the *L. paracasei* induced regulatory responses observed in this study. Stimulation of spleen cells from naïve wild type mice with *L. paracasei* induced production of IL-10 and TGF-β, and both IL-10 and TGF-β production were markedly reduced in TLR4^−/−^ derived splenocytes (Fig 7*A-B*). *L. paracasei*-stimulated splenocytes from TLR2^−/−^ mice had markedly impaired production of TGF-β while IL-10 production remained unchanged compared to wild-type splenocytes (Fig 7*A-B*). As expected, the production of cytokines triggered either by LPS (TLR4 ligand) or Pam3CSK4 (TLR2 ligand) was completely abrogated in spleen cells from TLR4^−/−^ or TLR2^−/−^ mice, respectively (Fig 7*A-B*). Thus, *L. paracasei* mediates the production of regulatory cytokines through TLR4 and partly through TLR2.

### Bone Marrow Derived Dendritic Cells May be an Important Source of Regulatory Cytokines Following Activation by *L. paracasei*

Dendritic cells (DC) are professional antigen presenting cells, which are involved in the polarisation of adaptive T cell immune responses (i.e. Th1, Th2 or regulatory). Stimulation of BM-DC with *L. paracasei* induced high levels of IL-10 and TGF-β in a dose-dependent manner (Fig 7*C-D*).

## Discussion

In this study we have shown that perinatal administration of *L. paracasei* NCC 2461 to pregnant/lactating mothers markedly attenuated allergic airway inflammation in their offspring. The protective effects were likely mediated by activation of regulatory pathways, signalling through TLR2 and/or TLR4 and were associated with suppression of both local and systemic immune responses.

Our findings are consistent with a recent clinical trial which showed that administration of a combination of probiotic strains to women during pregnancy and lactation without direct infant supplementation reduced the prevalence of eczema at 2 years [23]. The fact that direct infant intervention with probiotics may not be an absolute prerequisite for beneficial outcomes in offspring is an exciting observation, as there are considerable advantages in terms of convenience and compliance offered by maternal as compared to child-directed interventions.

There is a strong body of evidence suggesting a “window of opportunity” for modulation of infant immune responses during late gestation and the early perinatal period [13], [24], [25]. Indeed, farm exposure during pregnancy (as compared to postnatal farm exposure) was associated with the strongest protective effect against development of allergy [26]. We took advantage of this early “window of opportunity” and proved that intervention with *L. paracasei* during the process of maturation of immune system leads to anti-allergic phenotype in offspring. Nevertheless, our experimental approach does not allow us to distinguish whether the *L. paracasei*-driven effects were due to the prenatal intervention alone or to the combination of prenatal and early postnatal treatment.

Recently, it has been shown that prenatal maternal exposure to *Acinetobacter lwoffii* F78 prevented the development of asthma in the progeny which was associated with changes in H4 acetylation at the *IFNG* promoter [27], [28], [29]. Our findings support the intriguing possibility that stimulation of the maternal immune system during pregnancy with specific probiotic strains may generate signals that induce reprogramming of foetal immune responses, thereby protecting the offspring from development of allergic disease.

We observed that reduced airway inflammation in offspring after aerosol challenge with birch pollen was independent of changes in antibody titres in serum, which were not reduced by maternal exposure to *L. paracasei*. This situation is similar to human studies, where combined prenatal/postnatal probiotic treatment had no effect on atopic sensitization despite conferring a reduced prevalence of eczema (reviewed by Tang *et al.* [13]). Furthermore, our observations are in agreement with a study published by Blümer et al., where perinatal *Lactobacillus rhamnosus* GG (LGG) supplementation suppressed allergic airway inflammation in offspring without having an impact on allergen-specific immunoglobulin titres in serum [30]. Similarly, as described in our study with *L. paracasei*, *L. rhamnosus* primarily induces IL-10 production *in vitro* [31]. On the other hand, other probiotic strains, such as *Lactobacillus plantarum*, *Lactococcus lactis* or *Lactobacillus casei* strain Shirota, eliciting the induction of proinflammatory cytokines *in vitro*, have been shown to reduce allergen induced IgE-dependent basophil degranulation or directly inhibit IgE production in sera [32], [33]. In these cases the reduction of allergic immune responses is associated with a shift from typical Th2-based allergic responses to Th1-type immune responses. These observations indicate the complex character of probiotic applications which precise mechanism of action has to be still investigated as well as the clinical consequences.

Furthermore, perinatal *L. paracasei* treatment increased the production of gut IgA in offspring. Similarly, we have shown previously that intranasal application of probiotic bacteria enhanced production of secretory IgA in BAL of adult mice [14], [15]. Secretory IgA might act as “blocking antibody” by capturing and neutralizing of the allergens. Recently, Smits *et al.* proved elegantly that secretory IgA plays a prominent role in preventing the onset of allergic inflammation in a mouse model [21]. In a clinical trial, daily consumption of probiotic bacteria induced beneficial effects on allergic responses which were accompanied by a significant increase in mucosal IgA [34]. However, the importance of increased intestinal IgA in our model remains to be investigated.

With respect to cellular responses, the production of both Th2 (IL-4, IL-5) and Th1 (IFN-γ) cytokines was markedly reduced in re-stimulated spleen cells by maternal *L. paracasei*-treatment. This data are in agreement with our previous work, where the application of *L. paracasei* to the nasal mucosa led to reduction of allergen-specific Th2 responses as well as of IFN-γ in re-stimulated spleen cells [15]. This broad suppression in T cell responsiveness might be an explanation for the observed anti-inflammatory effects in the airways of offspring in the present study.

Surprisingly, maternal *L. paracasei*-treatment reduced production of IL-10 by allergen-re-stimulated splenocytes from offspring. This phenomenon was also observed in our previous study, where mucosal application of *L. paracasei* in a model of allergic poly-sensitization reduced IL-10 production in splenocytes [15], while allergic poly-sensitization and airway challenge itself was associated with increased IL-10 production. We therefore hypothesise that IL-10 is produced as part of a compensatory mechanism to control allergic inflammation, and so may be reduced following *L. paracasei* treatment if there is associated abrogation of allergic inflammation.

Notably, perinatal application of *L. paracasei* was associated with suppression of both antigen-specific and mitogen-induced responses in offspring. It remains important to evaluate whether intervention with certain probiotic strains might interfere with active immune responses against infections or vaccines. Even though such an effect has not been reported in published clinical trials, the question deserves to be evaluated in detail and may lead to additional selection criteria for strains destined to perinatal interventions.

De Roock *et al*. have shown that probiotic bacteria can induce functional Treg cells *in vitro* and block effector T cells function [35]. We have shown that stimulation of spleen or BM-DC with *L. paracasei in vitro* induced high levels of IL-10 and TGF-β, and that *L. paracasei*-induced production of regulatory cytokines by splenocytes is mediated via TLR2 and/or TLR4. Interestingly, certain probiotic bacteria have been shown to prime DC to promote the development of Treg cells [36]. Although we have not directly explored this possibility, it is attractive to postulate that the beneficial effects of perinatal *L. paracasei* might, at least in part, be explained by the induction of Treg cells through *L. paracasei*-induced regulatory DC. There is a body of evidence suggesting that the TLR ligands play an important role in prevention of allergic disease [37], [38]. Our data clearly demonstrates that TLR2 and TLR4 are important for recognition of *L. paracasei* and signalling through these receptors leads to induction of regulatory cytokines. In support of this, Conrad *et al.* have shown that maternal perinatal exposure to cowshed-derived bacteria induced resistance to allergic disease in offspring and functional maternal TLR-signalling was prerequisite for the transmission of protection [39].

In summary, our data demonstrate that maternal interventions by way of probiotic supplementation during gestation and the early postnatal period can modulate infant immune responses and thereby protect against unwanted allergic inflammation in the infant. This approach to disease prevention avoids direct interventions to the child, while having the capacity to influence regulation of immune responses in the infant.

## References

[1] Moffatt MF, Kabesch M, Liang L, Dixon AL, Strachan D (2007). Genetic variants regulating ORMDL3 expression contribute to the risk of childhood asthma.. Nature.

[2] Vercelli D (2008). Discovering susceptibility genes for asthma and allergy.. Nat Rev Immunol.

[3] Savino F, Cresi F, Pautasso S, Palumeri E, Tullio V (2004). Intestinal microflora in breastfed colicky and non-colicky infants.. Acta Paediatr.

[4] Savino F, Roana J, Mandras N, Tarasco V, Locatelli E (2011). Faecal microbiota in breast-fed infants after antibiotic therapy.. Acta Paediatr.

[5] Bjorksten B, Naaber P, Sepp E, Mikelsaar M (1999). The intestinal microflora in allergic Estonian and Swedish 2-year-old children.. Clin Exp Allergy.

[6] Kalliomaki M, Kirjavainen P, Eerola E, Kero P, Salminen S (2001). Distinct patterns of neonatal gut microflora in infants in whom atopy was and was not developing.. J Allergy Clin Immunol.

[7] Suzuki S, Shimojo N, Tajiri Y, Kumemura M, Kohno Y (2007). Differences in the composition of intestinal Bifidobacterium species and the development of allergic diseases in infants in rural Japan.. Clin Exp Allergy.

[8] Penders J, Thijs C, van den Brandt PA, Kummeling I, Snijders B (2007). Gut microbiota composition and development of atopic manifestations in infancy: the KOALA Birth Cohort Study.. Gut.

[9] Bisgaard H, Li N, Bonnelykke K, Chawes BL, Skov T (2011). Reduced diversity of the intestinal microbiota during infancy is associated with increased risk of allergic disease at school age. J Allergy Clin Immunol..

[10] Bjorksten B, Sepp E, Julge K, Voor T, Mikelsaar M (2001). Allergy development and the intestinal microflora during the first year of life.. J Allergy Clin Immunol.

[11] Holt PG, Upham JW, Sly PD (2005). Contemporaneous maturation of immunologic and respiratory functions during early childhood: implications for development of asthma prevention strategies.. J Allergy Clin Immunol 116: 16–24; quiz 25.

[12] Taussig LM, Wright AL, Holberg CJ, Halonen M, Morgan WJ (2003). Tucson Children’s Respiratory Study: 1980 to present.. J Allergy Clin Immunol 111: 661–675; quiz 676.

[13] Tang ML, Lahtinen SJ, Boyle RJ (2010). Probiotics and prebiotics: clinical effects in allergic disease.. Curr Opin Pediatr.

[14] Daniel C, Repa A, Wild C, Pollak A, Pot B (2006). Modulation of allergic immune responses by mucosal application of recombinant lactic acid bacteria producing the major birch pollen allergen Bet v 1.. Allergy.

[15] Schabussova I, Hufnagl K, Wild C, Nutten S, Zuercher AW (2011). Distinctive anti-allergy properties of two probiotic bacterial strains in a mouse model of allergic poly-sensitization.. Vaccine.

[16] Schwarzer M, Repa A, Daniel C, Schabussova I, Hrncir T (2011). Neonatal colonization of mice with Lactobacillus plantarum producing the aeroallergen Bet v 1 biases towards Th1 and T-regulatory responses upon systemic sensitization.. Allergy.

[17] von der Weid T, Bulliard C, Schiffrin EJ (2001). Induction by a lactic acid bacterium of a population of CD4(+) T cells with low proliferative capacity that produce transforming growth factor beta and interleukin-10.. Clin Diagn Lab Immunol.

[18] Wagner A, Forster-Waldl E, Garner-Spitzer E, Schabussova I, Kundi M (2009). Immunoregulation by Toxoplasma gondii infection prevents allergic immune responses in mice.. Int J Parasitol.

[19] Winkler B, Baier K, Wagner S, Repa A, Eichler HG (2002). Mucosal tolerance as therapy of type I allergy: intranasal application of recombinant Bet v 1, the major birch pollen allergen, leads to the suppression of allergic immune responses and airway inflammation in sensitized mice.. Clin Exp Allergy.

[20] Lutz MB, Kukutsch N, Ogilvie AL, Rossner S, Koch F (1999). An advanced culture method for generating large quantities of highly pure dendritic cells from mouse bone marrow.. J Immunol Methods.

[21] Smits HH, Gloudemans AK, van Nimwegen M, Willart MA, Soullie T (2009). Cholera toxin B suppresses allergic inflammation through induction of secretory IgA. Mucosal Immunol..

[22] Corthesy B, Gaskins HR, Mercenier A (2007). Cross-talk between probiotic bacteria and the host immune system.. J Nutr.

[23] Dotterud CK, Storro O, Johnsen R, Oien T (2010). Probiotics in pregnant women to prevent allergic disease: a randomized, double-blind trial.. Br J Dermatol.

[24] Feleszko W, Jaworska J, Rha RD, Steinhausen S, Avagyan A (2007). Probiotic-induced suppression of allergic sensitization and airway inflammation is associated with an increase of T regulatory-dependent mechanisms in a murine model of asthma.. Clin Exp Allergy.

[25] Osborn DA, Sinn JK (2007). Prebiotics in infants for prevention of allergic disease and food hypersensitivity.. Cochrane Database Syst Rev.

[26] Schaub B, Liu J, Hoppler S, Schleich I, Huehn J (2009). Maternal farm exposure modulates neonatal immune mechanisms through regulatory T cells.. J Allergy Clin Immunol 123: 774–782 e775.

[27] Brand S, Teich R, Dicke T, Harb H, Yildirim AO (2011). Epigenetic regulation in murine offspring as a novel mechanism for transmaternal asthma protection induced by microbes. J Allergy Clin Immunol..

[28] Renz H, Conrad M, Brand S, Teich R, Garn H (2011). Allergic diseases, gene-environment interactions.. Allergy.

[29] Renz H, von Mutius E, Brandtzaeg P, Cookson WO, Autenrieth IB (2011). Gene-environment interactions in chronic inflammatory disease.. Nat Immunol.

[30] Blumer N, Sel S, Virna S, Patrascan CC, Zimmermann S (2007). Perinatal maternal application of Lactobacillus rhamnosus GG suppresses allergic airway inflammation in mouse offspring.. Clin Exp Allergy.

[31] Latvala S, Miettinen M, Kekkonen RA, Korpela R, Julkunen I (2011). Lactobacillus rhamnosus GG and Streptococcus thermophilus induce suppressor of cytokine signalling 3 (SOCS3) gene expression directly and indirectly via interleukin-10 in human primary macrophages.. Clin Exp Immunol.

[32] Matsuzaki T, Yamazaki R, Hashimoto S, Yokokura T (1998). The effect of oral feeding of Lactobacillus casei strain Shirota on immunoglobulin E production in mice.. J Dairy Sci.

[33] Repa A, Grangette C, Daniel C, Hochreiter R, Hoffmann-Sommergruber K (2003). Mucosal co-application of lactic acid bacteria and allergen induces counter-regulatory immune responses in a murine model of birch pollen allergy.. Vaccine.

[34] Martinez-Canavate A, Sierra S, Lara-Villoslada F, Romero J, Maldonado J (2009). A probiotic dairy product containing L. gasseri CECT5714 and L. coryniformis CECT5711 induces immunological changes in children suffering from allergy.. Pediatr Allergy Immunol.

[35] de Roock S, van Elk M, van Dijk ME, Timmerman HM, Rijkers GT (2010). Lactic acid bacteria differ in their ability to induce functional regulatory T cells in humans.. Clin Exp Allergy.

[36] Smits HH, Engering A, van der Kleij D, de Jong EC, Schipper K (2005). Selective probiotic bacteria induce IL-10-producing regulatory T cells in vitro by modulating dendritic cell function through dendritic cell-specific intercellular adhesion molecule 3-grabbing nonintegrin.. J Allergy Clin Immunol.

[37] Bortolatto J, Borducchi E, Rodriguez D, Keller AC, Faquim-Mauro E (2008). Toll-like receptor 4 agonists adsorbed to aluminium hydroxide adjuvant attenuate ovalbumin-specific allergic airway disease: role of MyD88 adaptor molecule and interleukin-12/interferon-gamma axis.. Clin Exp Allergy.

[38] Velasco G, Campo M, Manrique OJ, Bellou A, He H (2005). Toll-like receptor 4 or 2 agonists decrease allergic inflammation.. Am J Respir Cell Mol Biol.

[39] Conrad ML, Ferstl R, Teich R, Brand S, Blumer N (2009). Maternal TLR signaling is required for prenatal asthma protection by the nonpathogenic microbe Acinetobacter lwoffii F78.. J Exp Med.

